# Should We Consider Alternatives to Universal Well-Child Behavioral-Developmental Screening?

**DOI:** 10.3389/fped.2015.00021

**Published:** 2015-03-17

**Authors:** Jacob Urkin, Yair Bar-David, Basil Porter

**Affiliations:** ^1^Division of Community Health, Faculty of Health Sciences, Ben-Gurion University of the Negev, Beersheba, Israel; ^2^Division of Pediatrics, Soroka University Medical Center, Beersheba, Israel; ^3^Clalit Health Services, Tel Aviv, Israel

**Keywords:** child, development, screening, surveillance, well-child-care

## Abstract

The prevalence of developmental disabilities in the young age is of the order of 15%. When behavioral and social-emotional disorders, physical impairments, and sensory disorders are included, the need for special intervention increases to one out of four children. As the sensitivity and specificity of the best screening tests are in the range of 70–80%, their predictive value is controversial. The cost of conducting definitive tests and repeat screening for those who fail the screening tests is high. Children with severe disorders can be identified clinically without a screening test. The poor predictability, difficulty in implementation, and the high costs of developmental testing suggest that children, particularly those in high-risk communities, might be better served by implementing intervention programs for all, instead of trying to identify the outliers through screening.

## Introduction

At least 10% of children have some degree of behavioral-developmental disabilities (1). Much higher rates are quoted in *Nelson Textbook of Pediatrics* (2). These include 17.5% of children with speech-language impairment, up to 14% with social-emotional disorders, about 8% of children who have attention deficit/hyperactivity disorder, 1% with autistic spectrum disorder, and 6.5% with learning disabilities. In addition, there are children with vision/hearing impairments, physical impairments, and intellectual disabilities. Many children have more than one problem. It seems that the actual prevalence of developmental behavioral problems in the pediatric age may be as high as 20% (3–5). The burden of identifying and managing these children is shared by both educational and health services. Based on clinical impression alone, physicians fail to detect most children with behavioral-developmental problems (6). The American Academy of Pediatrics and many health agencies worldwide recommend developmental surveillance and screening as a tool for detecting developmental-behavioral disabilities (7, 8). Early intervention at a young age following early detection by screening carries the promise of a reduction in the risk of school failure, school dropouts, and social malfunction (2).

Although numerous screening tests have been conducted over the years in many settings, only a few have demonstrated clear long-term benefits. Neonatal hearing screening is a good example. The American Academy of Pediatrics “Bright Futures” program states that most screening tests and interventions lack good research to support the recommendations (8). This does not mean that they are ineffective, since screening is able to find children and families who are at risk and to initiate the cascade of testing and interventions they need. This strategy targets some children but does not help the rest. Programs such as the “Incredible Years Program,” “Reach Out and Read,” and “Triple P” took another approach by intervening in communities without the use of a screening test (9–11). Triple P is a parenting program demonstrating that by improving parenting skills child development and child–parent interaction are enhanced (10). The Incredible Years Program coaches parents on how to deal with children’s behavioral problems and promote their social, emotional, and academic competencies (9). These programs were shown to enhance child development and child–parent interaction, and to improve child behavior in a diverse range of families (9, 10). The Reach Out and Read program encourages parents to read books to babies and toddlers. Children in the program demonstrated significantly higher vocabulary and language skills when compared to children who were not in the program (11). Some of these programs are limited by addressing only a narrow band, but not the whole spectrum of children’s problems and developmental needs, thus they may not replace the need for broad-spectrum developmental screening.

The justification for developmental screening is based on the premise that health and development are interrelated. The plethora of screening tests reflects the difficulty in developing a single test that is significantly better than others. The questions are: does developmental/behavioral screening fulfill the epidemiologic criteria for effective screening tests? Is the prevalence of the condition common enough to justify universal screening? Is the screening truly universal? Do screening and the resulting interventions promise better outcomes? Is screening for developmental disabilities cost-effective? Does it have high positive and negative predictive values, i.e., high rate of identification of the children with a real problem?

This paper does not purport to be a review of this important subject, but rather to challenge some of these premises and to reconsider the justification and alternatives to universal behavioral-developmental screening.

## The Relationship between Health and Development

Health and nutrition are prerequisites for optimal development (12, 13). The relationship between nutrition and development was highlighted in studies on iron deficiency and its influence on psychomotor and social development (14). Malnutrition for prolonged periods of time is associated with developmental delay (15). Micronutrient and vitamin deficiencies may lead to growth and developmental deficiencies. Iodine and thiamine deficiency are well-documented causes of developmental delay (16).

However, nutrition is only one factor influencing development. Genetic disorders causing congenital metabolic diseases, brain dysplasia, or other congenital malformations are well-known factors that adversely affect health and development. However, these cases represent a minority of children with behavioral-developmental needs. For most, nurture is, by far, the most influential factor that determines health and development. Well-child-care, in its modern form, emphasizes nurture in two ways. First, by incorporating knowledge about optimal nutrition, exemplified by the promotion of breast feeding and recommendations for infant formulas with constituents that are as close as possible to mother’s milk. Second, by teaching parents how to interact with their children and to provide the right environment for stimulating development (15).

The first years of life provide a window of opportunity in which the structure and the foundation of the functioning brain are established. Failure to provide optimal health and developmental stimulation in the early years of life may have serious implications throughout the life course (17).

## Is the Screening Truly Universal?

The success of screening, as a method of identifying a sub-population with a high risk of having an abnormal condition, depends on the ability to implement universal screening. Well-child-care services provide an opportunity to conduct screening tests on a “captive audience” attending the clinic. However, the ability of well-child-care services to capture the entire target population is not guaranteed. Mothers may not attend well-child-care clinics for a screening test for their babies without having an additional incentive. One of the main incentives is the provision of free immunizations (18). The time when polio, whooping cough, and meningitis were common devastating childhood illnesses is still recent enough to encourage most people to immunize their children. Unfortunately, the success of immunizations in eradicating severe childhood diseases has led some parents to believe that concern for these diseases is obsolete, leading to a decline in immunization rates, with a consequent decline in attendance at well-child clinics and failure of these children to undergo developmental screening.

In addition to immunizations, mothers are expected to bring their babies to a well-child clinic for nutritional consultation and guidance, monitoring of physical growth, and developmental surveillance (19). Their concern regarding these issues depends on their experience in childrearing, family values, and the influence of health care providers and the media. These do not suffice to ensure high rates of attendance. The geographic distributions of health care facilities may influence accessibility and parental willingness to invest the time to attend well-baby clinics. A fee for developmental screening acts as a deterrent to universal screening.

One may take the position that screening some of the population is better than not screening at all. However, when screening rates drop below a critical level their value as a universal tool to detect children at risk is lessened. Moreover, the population that is not screened might be the very population with special risk factors for behavioral-developmental disorders. Poverty, social problems, low parental education, and parental psychiatric problems are risk factors for special developmental needs as well as for non-attendance for preventive services (20). Relying on a system that is declared open to all but actually serves only part of the population might miss those most in need.

## Do Screening and the Resulting Interventions Promise Better Outcomes?

This is, indeed, a disputable issue; the literature shows studies that both support and controvert this idea (17, 21–25). However, even when early intervention is proven effective it may not necessarily be the result of an early screening test, but rather of alternative paths for identifying children at risk.

There are at least three issues that highlight the difficulty in studying the effectiveness of early intervention. Development is a continuous process that is affected by multiple factors. Early diagnosis and intervention are not the only modifiers that determine outcome. As every baby is unique, biologically and in its environment, it is almost impossible to evaluate the relative influence of intervention on his/her developmental outcome, i.e., is the child improving over time despite or because of the intervention? As we cannot reverse time, there is no research method that can evaluate the influence of different interventions on the same child starting at the same point in time.

When groups of children are evaluated by comparing two or more interventions, there are studies that strongly support early intervention (26, 27). These studies should be judged very cautiously. In disorders that could stem from many different factors, for example, speech delay that could stem from an undiagnosed mild hearing impairment, low-average intelligence, or insufficient language interactions with others, it is difficult to match the subjects, necessitating some compromises as the groups are not identical but only similar. In addition, such studies are not blinded, at least to the parents and the staff. In many interventions, the evaluation of the outcome will take place many years after the start of the intervention while other non-matched factors are intervening during that period. These issues may lead to bias and exaggeration of the value of early intervention.

In addition, researchers and journals tend to publicize studies that show significant differences in favor of an intervention, possibly leading to publication bias. When an intervention shows no added value, the chance of publication is usually low.

Another issue is an ethical one. In a society where early intervention is strongly endorsed, researchers will not design a study in which one arm of the study includes a “no intervention” group. Under such circumstances, we may never know whether no intervention is as good as any intervention. This methodological difficulty could be resolved if parents randomly refuse intervention or in places where intervention is randomly unavailable for all. Such randomizations are rare in the real world.

A third issue concerns vested interests. Any health system is an industry that needs to maintain its survival and economic stability. Many professionals and non-professionals are involved in well-child care, conducting screening and diagnostic testing, and providing a range of therapeutic and rehabilitation interventions. There is thus an economic incentive to maintain and increase the volume of activities by emphasizing the benefits of early screening and intervention and minimization of doubts about outcomes. Moyer and Butler reviewed the literature on screening recommendations of seven major North American health care organizations. Of 21 screening recommendations that were endorsed by at least two agencies, behavioral and developmental assessments did not have evidence-based support of effectiveness (28).

Non-medical healers from alternative or complementary medicine promise tests and treatments for developmental problems by methods that are not scientifically proven, adding to the notion that screening is justified.

A practical issue further challenges the premise that screening tests are the way to ensure better outcomes for the general population of children. Many physicians administer standard screening tests in a non-standard manner or do not use structured screening tests at all (29, 30). Poor compliance by parents and poor follow-up by health providers add to the missed opportunities for successful early intervention (29, 31). These practical issues challenge the wisdom of governmental and professional organizations endorsing the importance of screening (32).

## Is Screening for Developmental Disabilities Cost-Effective – Are There Alternatives?

By definition, a screening test should be relatively cheap, easy to perform, simple, and acceptable. The long list of available behavioral-developmental screening tests is confusing. Some require a long period of time to perform and score and thus are costly (33, 34).

More costly are the tests and consultations that are needed to verify or to exclude a diagnosis, which are carried out on children who test positive on the screening test. A screening test with a high rate of false positives will thus be very costly. As even the best screening tests have a sensitivity and specificity of 70–80%, about 25% of the population will be included in this category.

A screening test with a low false positive rate is also risky as more children with a real problem will be screened as false negatives. These children slip through the net of the screening test and their parents enjoy a false sense of security that their child does not have a developmental disorder. Almost certainly, in these children there will be a delay in diagnosis and subsequent intervention. Late diagnosis increases the chance of preventable complications, misses opportunities to prevent more births with the same genetic problem, and results in higher medical and non-medical expenditures. The cost assessment should also be compared to the alternative, which is the cost of managing the cases, whenever they are diagnosed, when screening is not conducted.

The most difficult part in the equation is the evaluation of the effectiveness of screening tests. What is the outcome that we would like to measure and can it be translated into measurable scales? Are we looking for length of life, number of survivors, quality of life, school achievements, or level of functionally independent life? In disorders where outcome of intervention will be judged many years after the time of screening, it is almost impossible to evaluate whether outcome is related to a screening test.

Dobrez et al. estimated that the cost per month per child for 0- to 3-year-old children for screening tests is $4 to $7 (34). Assuming a cost of only $5, the cost of screening alone will be $60,000 per year for 1000 children.

Regarding universal intervention, in a study by Aos et al. every $1 invested in the Triple P system yielded a $9 return in terms of reduced costs of children in the welfare system (35). A comparison of the economic return for many general intervention programs that aim at enhancing children’s development and family functioning was summarized by the WAVE Trust organization in the UK (36). The benefit to cost ratio, which presents the ratio between the values, in monetary terms, gained from running a program to the amount of money that was invested, ranged between 4.2 and 7.5 in the Incredible Years programs. The benefit to cost ratio of the Triple P program is 9.22. UK social return showed returns between £1.37 and £9.20 for every £1 invested in universal parenting programs in the first 2 years of life.

Compliance with screening recommendations is a problem by itself. Even when the money is allocated, funded court-ordered mandated early and periodic screening, diagnostic, and treatment (EPSDT) screening for mental health problems during Medicaid well-child visits was not conducted in almost 50% of the target population (37). The conclusion is that screening is costly and partially done and the evidence about the ratio of benefit to cost from interventions is high. Thus universal intervention is suggested as a better investment.

In regard to child development, it might be wiser to spend the money on parental education, social welfare, child nutrition, and early childhood developmental stimulation (38). Such a policy might sacrifice a small number of borderline cases of developmental delay as the severely affected will be diagnosed early anyway, but will benefit a larger portion of the population who will be granted a head start in the form of better parenting, education, and environment in places where the trade-off might be justified.

## Do Developmental Screening Tests Provide Satisfactory Predictive Values?

There are studies that question the yield of developmental screening tests (5, 39–41). The best tests have sensitivity and specificity in the range of 70–80% (28–30). Are we satisfied with these figures? University professionals who have a special interest in human development usually develop screening tests; however, these tests are intended to be used by personnel who are generally less focused on human development and less educated on that subject. This gap carries the risk of “cutting corners” by inaccurate performance and scoring of tests (42). Although clinical-judgment-based developmental surveillance fails to identify about 50% of children with developmental problems, a validated screening test misses too many cases when implemented in clinical settings, thus reducing its predictive value (6, 43–47).

Standardized screening tests fail to adjust for the cultural differences between communities and the way children are raised. This is especially true for evaluation of social and communication skills, which may reach 100% failure in the screening test (48).

An additional point to be taken into account is the approach when a child’s performance is below average but within 2 SD for age. Half of the population is there. The approach will determine the decision regarding re-screening, follow-up, and further testing. The opinions of parents, teachers, and medical professionals are influenced by this issue. Many will vigilantly pursue further testing and interventions when a child performs below the average. The goal of raising a developmentally abnormal child has changed from one of raising a child who is developmentally normal to one who is above the average!

Many children who fail screening tests will pass the definitive tests. These children may still have low normal developmental skills and have social risk factors for developmental delay. They will benefit from early universal developmental interventions and the earlier the better (17, 49). We do not need screening tests to do that.

Last but not least, many of the problems that screening tests are designed for are identified by parents, relatives, and health and educational personnel without a screening test (50). At least for severe and moderate cases, this would be expected to happen early, before, or about the time of the planned screening tests.

A screening test may cause harm by making parents more worried and unnecessarily labeling children, with no added value regarding the developmental prognosis. A study by Cadman et al. on 4797 preschool children demonstrated the ineffectiveness of the DDST screening test and questioned whether mass developmental screening tests do more harm than good (51). Blair and Hall are concerned about the risk of stigmatizing parents or criticizing parenting skills and thus suggest social educational augmented services for all (3). In some communities, all children should be regarded as at risk and for them screening tests are probably redundant (48). Proceeding directly to universal developmental interventions and investing in families and community might be the alternative. The latter suggestion is an alternative investment of public health funds where the emphasis is shifted from child health surveillance to child health promotion (3).

This notion is supported by research that demonstrates a low sensitivity of developmental screening tests and poor referral and compliance for those who are diagnosed with a problem (6, 52–54). A critical review by Moyer and Butler concluded that rigorous evidence supporting screening is very limited (28). A meta-analysis of the literature on speech and language delay in preschool children concluded that there are no data supporting the effectiveness of screening in a primary care setting (55). The US preventive services task force concluded that the evidence is insufficient to recommend for or against routine use of a formal screening instrument in primary care to detect speech and language delay in children up to 5 years of age (55).

Shifting from universal screening to diagnostic testing is already occurring in regard to developmental dysplasia of the hip. Babies are universally referred for imaging of the hip joint, even when the physical examination is normal. Sending all toddlers to the ophthalmologist to rule out strabismus or refractive disorders reflects the same trend. Parental awareness of behavioral-developmental problems and risk management issues is expected to further advance the trend for testing and skipping screening (56). A recently published algorithm for developmental-behavioral surveillance and screening demonstrates the complexity of screening and its management, and could further promote universal testing instead of screening (57).

The notion that parents are good observers of their child’s developmental abilities led to the development of screening tests that are based on structured questioning of parents (58). The Ages and Stages Questionnaire (ASQ), the Child Developmental Inventory, and the Parents’ Evaluations of Developmental Status (PEDS) are three prominent examples. Some researchers are less positive regarding the reliability of information provided by parents (59). These tests do not identify the same children, which may suggest that a child may need more than one screening test (60, 61). The burden of conducting more than one screening test will obviously be very costly, depending on how many additional tests are considered “justified” and sufficient. As developmental surveillance is a continuous process, this question will repeat itself. However, there is no study that has examined the consistency of screening tools used to track children over extended periods of time (59).

A cheaper and better alternative to a standardized screening test that is conducted in a medical setting might be to use the reports of kindergarten teachers (50, 62). These teachers observe the children over long periods of time and in a natural social environment. Teachers’ standardized reports could become an alternative behavioral-developmental screening tool (50). Teachers have the advantage of education on child development, relative objectivity, and experience with behavioral-developmental achievements of many children. This alternative has the ability to overcome parental literacy or psycho-emotional difficulties, reluctance to disclose information that may indicate abuse or neglect, and subjective non-realistic judgment of a child’s development. It will also relieve some of the burden imposed on medical services.

An expert group from Australia has scrutinized many screening tests in depth. Most of them were summarized as lacking justification for their continuance. In the executive summary it states that there is no high quality evidence putting together all the links in the chain and reporting on the effectiveness of developmental screening programs on child developmental outcomes (63). Others have reached similar conclusions (64).

## Conclusion

Screening for developmental-behavioral problems is endorsed by many professional organizations and practices as part of good clinical practice for the care of children. The current paper addresses the limitations of screening. Beyond screening, a variety of universal early interventions such as parenting interventions, Incredible Years, and Triple P have demonstrated efficacy in improving a range of child outcomes such as social skills and disruptive behavior. Some of the programs are limited by addressing only a narrow band of child developmental issues. We do not expect that early intervention programs will overcome the need for universal broad or narrow band screening. Screening should continue. However, we need better tailoring of our public health efforts and expenditures. We believe that there is room to re-think the issue of screening as the desired dominant path for early diagnosis and intervention for developmental problems. The poor predictability, the difficulty in implementation, and the high costs of developmental testing suggest that we might serve children better, particularly those in high risk communities, by implementing more intervention programs for all, instead of trying to identify the outliers through screening. The dangers of “missing” mild and minor developmental delay might be limited by careful and regular surveillance of children’s activities and their adjustment in daily activities. This could be achieved by training health and educational personnel who see the children on a regular basis. It would enable funneling more funds from screening to early intervention programs that have demonstrated effectiveness. Further research should target the new balance between screening and universal intervention.

## Author Contributions

JU: reviewed the literature, produced the first draft of the manuscript, and guided the discussions among the authors. YB-D: carried out the literature research, was active in the discussion, and contributed original concepts based on his field experience in well-child care services. BP: supervised the discussion on the manuscript, added his ideas based on his long career in well-child services as head of a child development center, and finalized this version of the manuscript.

## Conflict of Interest Statement

The authors declare that the research was conducted in the absence of any commercial or financial relationships that could be construed as a potential conflict of interest.
